# Unsaturated Response of Clayey Soils Stabilised with Alkaline Cements

**DOI:** 10.3390/molecules25112533

**Published:** 2020-05-29

**Authors:** Nuno Araújo, Manuela Corrêa-Silva, Tiago Miranda, António Topa Gomes, Fernando Castro, Tiago Teixeira, Nuno Cristelo

**Affiliations:** 1 ISISE, Department of Civil Engineering, University of Minho, 4800-058 Guimarães, Portugal; nuno@civil.uminho.com; 2 Department of Civil Engineering, University of Minho, 4800-058 Guimarães, Portugal; a61942@alumni.uminho.pt; 3 Institute of Science and Innovation for Bio-Sustainability (IB-S), Department of Civil Engineering, University of Minho, 4800-058 Guimarães, Portugal; tmiranda@civil.uminho.com; 4 CONSTRUCT, Department of Civil Engineering, University of Porto, 4200-465 Porto, Portugal; atgomes@fe.up.pt; 5 MEtRICs, Department of Mechanical Engineering, University of Minho, 4800-058 Guimarães, Portugal; fcastro@dem.uminho.pt; 6 W2V, Rua das Alminhas 900, 4810-608 Guimarães, Portugal; tteixeira@w2v.pt; 7 CQ-VR, Department of Engineering, University of Trás-os-Montes e Alto Douro, 5001-801 Vila Real, Portugal

**Keywords:** sustainable cement, alkaline activation, soil stabilisation, unsaturated soils, retention curves

## Abstract

The influence of suction on the mechanical behaviour of unsaturated chemically stabilised soils is still mostly unknown and unquantified. This is also motivated by the difficulties associated with the experimental procedure required to fully characterise the unsaturated response of the soil, including its direct influence on traditional strength tests. The present paper presents the soil water retention curves obtained for a Portuguese soil before and after being stabilised with Portland cement (OPC) and an alkali-activated cement (AAC). Saturated undrained triaxial tests were also performed for the same curing conditions (0, 28, and 90 days). Previous attempts to characterise the retention curve of soils stabilised with AAC are unknown, and the results showed that the pore volume structure is already formed after 28 days, prior to the full development of the gel matrix responsible for the strength increase between 28 and 90 days. The curve changed after stabilisation, and with each binder, as the OPC presented a higher air-entry value and a narrower suction range compared to the AAC solution. The significant differences between the curves obtained from each binder suggest the future development of a methodology to assess the quality of the AAC stabilisation.

## 1. Introduction

Soil improvement through the addition of chemicals is usually applied with the aim of enhancing the soil’s mechanical and physical performance. The most common binders are lime and Portland cement (OPC), which either react with the soil particles (the former) or simply form a cement structure around them (the latter). Up till recently, this was a relatively straightforward and effective process, with many decades of experience and a large volume of behavioural data collected over the years. However, the rising and ever more acute concerns with the environment have shifted the most important paradigm in the construction industry—the Portland cement dependency—towards the need for more environmentally friendly binders. Alkaline activation is a strong example which, by allowing the introduction of industrial wastes and residues into the fabrication of such alternative binders, represents a twofold advantage, by mitigating the need for OPC and by reducing the volume of wastes that are landfilled [1,2,3,4,5,6,7,8,9].

Alkaline activation (AA) frequently uses fly ash (FA), resulting from the combustion of coal in thermo-electric powerplants, as a precursor. This is because this particular FA not only possesses high contents of silicon and aluminium, both essential in the formation of an alkaline cement structure, but has also been submitted to high temperatures, during combustion of the coal, which removed most of its constituent water and, thus, significantly increased the degree of amorphization. This is an essential property of the precursor, as a crystalline material will be less available to respond to the activation reactions [10].

The application of this alternative technique in soil stabilisation results in a hardened gel surrounding the soil particles, with low interaction between the two, as the soil particles are essentially inert to the chemical activation of the precursor (e.g., fly ash, blast furnace slag, metakaolin) by the alkaline solution (usually sodium hydroxide and/or sodium silicate) [11]. Although it can be regarded as a more sustainable alternative to OPC, it depends heavily on wastes and alkaline materials, both of which could be somehow harmful to the environment. However, several studies have already shown that the resulting crystalline gel very effectively encapsulates all heavy metal ions and other potentially concerning leachable elements [12]. It has also proved to be very durable, clearly competing with OPC standards [13].

From a mechanical performance perspective, the first studies focusing on the use as a soil stabiliser have demonstrated clear and significant increases in compressive strength [2,3,14] and split tensile strength [15], as well as increases in stiffness [16,17], relatively to the original soils. The effectiveness of the technique is also a function of the intrinsic properties of the soil, but especially of the quality of the precursor (unburned rate, original composition, amorphisation degree), of the type of activator (alkali concentration, hydroxide/silicate ratio) and of the activator/precursor ratio (including the total water content and the alkali/precursor ratio) [1,16,18].

Also important, when designing soil stabilisation with alkaline cements, is that their strength gain rate might be lower than that usually seen with OPC. The latter reaches very significant increases after only 28 days (90 to 95% of its ultimate compressive strength), while alkaline cements based on low-calcium precursors, like the FA used in the present study, have shown significant strength gains even after 90 days [1,14]. The soil used during this research work was already submitted to previous studies that primarily focused on stabilisation with cement and lime, but also with alkaline cements based on FA, aiming at increasing its uniaxial compressive strength (UCS) [2,16]. In short, these studies found that conventional binders reached higher UCS values after 28 days, while the alkaline cements were able to produce higher UCS for longer curing periods, up to 90 days. The effectiveness of the alternative binder proposed, especially after 90 days, was confirmed by the higher stiffness presented, relatively to the OPC-stabilised soil. The present paper extends the previous mechanical characterisation work, by presenting triaxial tests on the stabilised soil, controlling suction, which might play a decisive role in the obtained results, using either OPC and alkaline cements, after 28- and 90-days curing.

Total suction (*ψ*) is the most relevant parameter controlling the behaviour of unsaturated soils, since it can significantly alter the soil’s response regarding its mechanical behaviour, including strength and stiffness [19,20,21], but also influencing air, water and heat flow, relevant variables regarding environmental problems [19,22]. Total suction corresponds to the sum of the osmotic suction and the matrix suction. The former refers to the movement of salts through the free water existing in the soil’s voids, caused by different salt concentrations, which can produce water movements; while the latter refers to the difference between atmospheric pressure and void water pressure [23], which is equal to the energy required to remove a water molecule from the soil structure [24]. In geotechnical engineering, total suction often corresponds to the matrix suction alone, i.e., osmotic suction is not considered [19,20], since free water is considered to be neutral. To fully characterise the hydromechanical behaviour of unsaturated soils, it is important to evaluate the matrix suction, as it is influenced by environmental conditions and can lead to changes in soil volume, shear strength and permeability [19,20,22,25].

The present study focused on the analysis of the unsaturated response of a sandy lean clay stabilised with AA cement (based on coal fly ash activated with sodium hydroxide and sodium silicate). This type of binder is a more environmentally friendly alternative to common Portland cement, which was also used in this study, to create a threshold. The study was mostly based on the retention curves and triaxial tests, before and after stabilisation, and after curing periods of 28 and 90 days.

## 2. Results

### 2.1. Soil Water Retention Curves (SWRC)

**Figure 1:**
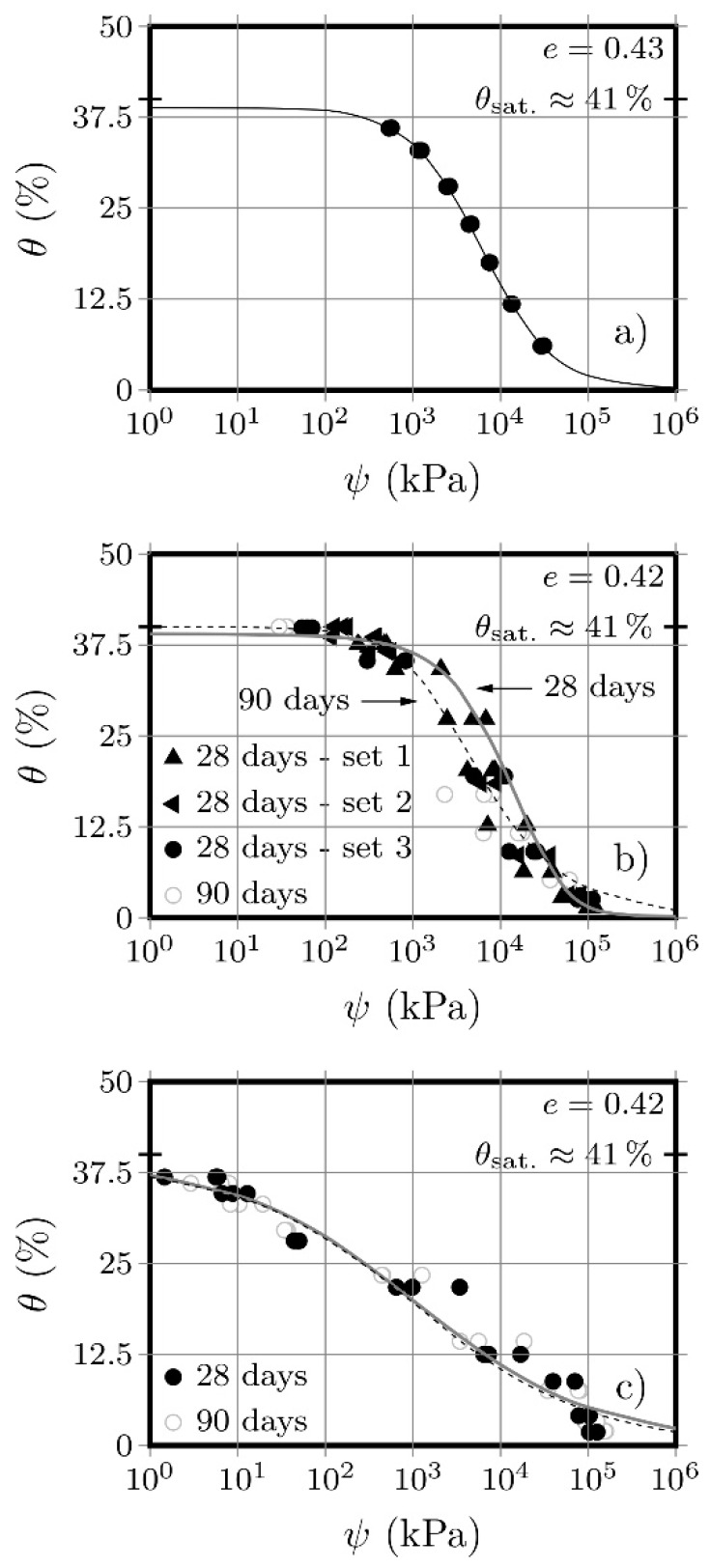
Experimental data and theoretical soil-water retention curves (using van Genuchten proposal) for the original soil (**a**), for the Portland cement-soil combination (OPC-S) material (**b**) and for the alkali-activated cement-soil combination (AAC-S) material (**c**) (Note 1: the values obtained with each of the 3 paper filters used are included; Note 2: three sets of tests were developed for 28-days OPC-S material; Note 3: the theoretical saturation value of the original soil was estimated at 41%, and used to fit the curves).

The matrix suction (*ψ*) as a function of the volumetric water content (i.e., retention curve) is shown in Figure 1, for the original soil (a) and for the soil stabilised with AAC (b) and OPC (c). The results show a suction increase with the decrease of the water content for all materials, which is a consequence of the negative net-pressure between the saturated and the residual unsaturated soil areas. It is in the transition zone that the suction forces, responsible for altering the mechanical behaviour of the material, are developed. The range of suction values in the original soil is clearly shorter than that obtained for the stabilised materials, especially when compared with the AAC-S material. Furthermore, the air-entry value presented by the soil (approximately 700 kPa), is abnormally high, demonstrating that it is almost insensible to suctions up to this value.

The artificial cementation causes an increase in the soil’s ability to retain water, preventing a rapid decrease between the saturated state and the residual unsaturated state. The saturation volumetric water content (*θ_s_*) obtained for the original soil was 39.4%, slightly lower than the theoretical value of 41%), while the residual volumetric water content (*θ_r_*), from which no water is further extracted from the soil, was 0%. The OPC-S material presented similar saturation volumetric water content values after 28 days (*θ_s_* = 39.4%) and 90 days curing (*θ_s_* = 40.1%). The residual volumetric water content values, after 28- and 90-days curing, were 0%. Very similar results were obtained with the AAC-S material, with saturation water content values equal to *θ_s_* = 39.4%, either after 28 days or 90 days curing; and residual water content values equal to *θ_r_* = 0%, after both curing periods. The fact that the *θ_r_* value of 0% obtained for the original soil was kept constant after stabilisation (with either binder), indicates that the pore structure of the material maintained its capacity to remove all water under high suctions. Such low *θ_r_* in the stabilised material could indicate that some osmotic suction developed during the curing process, but the original soil followed the same trend.

Another relevant aspect is the air entry value obtained for each material. While the natural soil registered 700 kPa, the stabilised soil registered approximately 100 kPa (OPC-S) and 10 kPa (AAC-S). The reasons behind such a difference need to be further studied. However, considering that the air entry value depends mainly on the pore size distribution [19] it is clear that the artificial cementation of the soil particles had significant consequences on its size distribution. Nevertheless, the different air entry values showed by each soil-stabilised material (i.e., the AAC-S initial value is only 10% of the OPC-S) and the significantly different shape of the SWRC of both stabilised materials, suggests that each stabiliser influenced the particle size distribution and, more importantly, the pore size distribution, in a very specific and different way. It is well known that a wider range of pore size produces a smoother suction variation with water content, while a uniform pore size distribution is responsible for more abrupt suction variations [26,27]. Based on the higher suction variation of the AAC-S material, it can be assumed that this binder produced a wider pore size variation, either due to its own composition (not all fly ash particles are ‘attacked’ during the chemical reaction that produces the binding gel, and thus remain as part of the solid skeleton), or to the interaction between the binder and the soil’s particles.

The OPC-S material presented an intermedium transition rate, from the saturated to the unsaturated residual phase, between that of the original soil and the AAC-stabilised material, represented by a suction range starting at approximately the average value of the previous two materials. The results do not show a marked variation between the results obtained with the 28- and 90-days curing either in terms of the saturation volumetric water content (*θ_s_* = 30.4% and 30.1%, after 28 and 90 days curing, respectively) and the residual volumetric water content (*θ_r_* = 10.4% and 10.5%, after 28 and 90 days curing, respectively). The stabilization with Portland cement produces a different structure, relatively to the original soil and even to the AAC-stabilised soil. Regardless of the more homogeneous binding gel (C-H-S) distribution, the chemical process obtained from cement Portland is less interactive with the surface of the soil particles, resulting in a more homogeneous pore volume distribution than that showed by the AAC treated material.

The AAC-S material showed a smoother transition between the saturated and unsaturated residual phase than either the original soil or the OPC-S material, while also presenting a higher range of suction values. This behaviour originates from the new soil structure, formed under the influence of the aluminosilicate gel that resulted from the activation of the fly ash particles, resulting in a more compact matrix that includes the unreacted particles [28]. The formation of such gel leads to pore segmentation, resulting in a structure similar to that of a natural rock, in molecular terms [14]. No significant differences were detected as a result of the different curing periods of the specimens, which was confirmed either by the similar saturated volumetric water content values (*θ_s_* = 39.4%, after 28 and 90 days curing), and the residual volumetric water content values (*θ_r_* = 0%). Given the evolution of the aluminosilicate gel over time [28], some influence of the curing time on the matrix suction was expected. However, these results suggest that, based on the complexity of the gel formation process (dissolution, condensation, precipitation, crystallisation), the overall gel matrix is already formed after 28 days, and only the last stage (i.e., crystallisation) is yet to be fully developed. Furthermore, these results suggest also that such gel crystallization had no influence whatsoever in the pore structure of the material or was responsible for any osmotic-type suction. Therefore, the soil-gel system is not modified after an early stage of the gel development. This is corroborated by the strength tests’ data presented ahead, showing that the AAC-S material was indeed more performing after 90 days than after 28 days, in terms of mechanical behaviour, which results from the hardening (crystallization) of the binding gel structure.

### 2.2. Triaxial Tests

**Figure 2:**
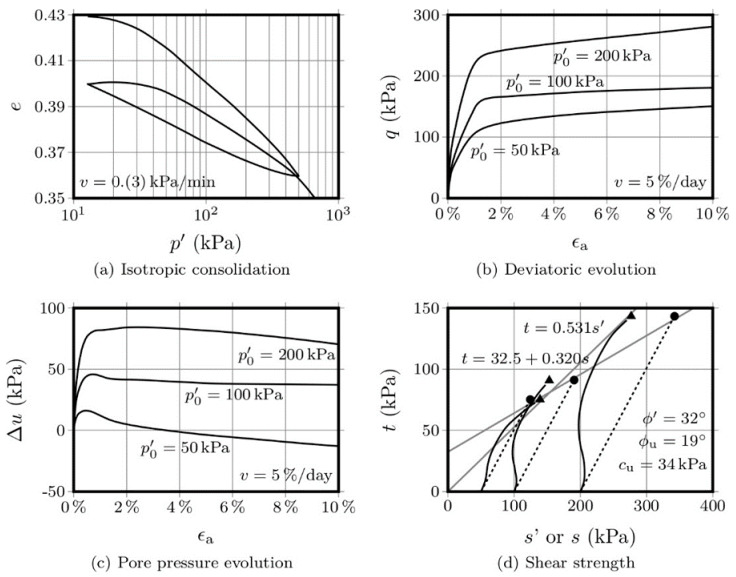
Consolidated undrained triaxial tests performed on the original soil (without artificial cementation).

The behaviour evolution of both the original and stabilised soil, obtained through undrained consolidated triaxial (CU) tests, is presented from Figure 2a to Figure 2d. Figure 2a presents the original soil under isotropic compression, namely the variation of the voids ratio (Δ*e*) as a function of the effective mean stress (*p*’). It is clear that the variation in Δ*e* is higher than that presented by the chemically stabilised materials, for the same applied mean effective stress, reinforcing the idea that stabilisation decreases deformability of the native material. Figure 2b presents the stress-strain curve, i.e., the deviatoric stress (*q*) as a function of the axial strain (*ε_a_*), where it is possible to observe that samples with a higher consolidation stress present higher stiffness. No peak stress was observed for this untreated material, which followed a strain-hardening stress-strain path, by still allowing a stress increase with increasing strain, a clear indication of its original ductility and high-deformation capacity [29]. The pore pressure variation (Δ*u*) is also presented in Figure 2c, showing a rapid increase at the start of the test, which stabilises after yielding. Finally, Figure 2d presents the stress path in s–t coordinates, from which the effective friction angle (*φ*’ = 32°) and the undrained friction angle (*φ_u_* = 19°) were estimated, as well as the undrained cohesion (*c_u_* = 34 kPa).

**Figure 3:**
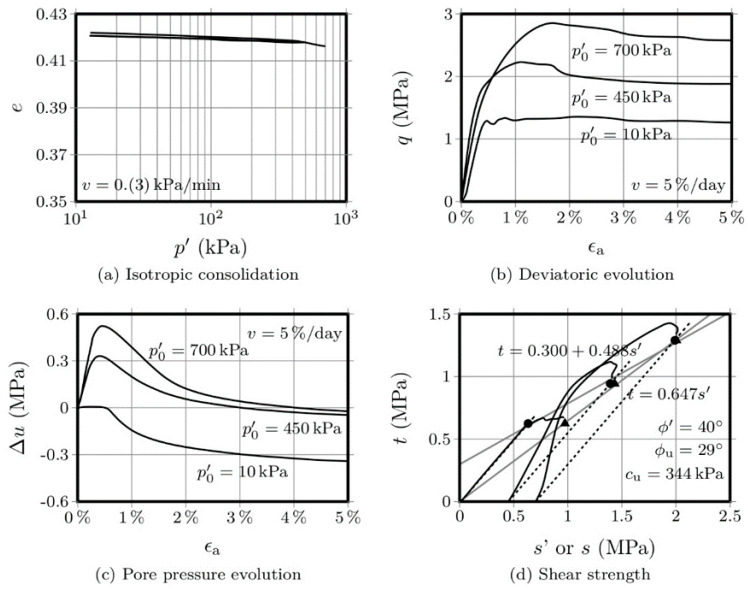
CIU triaxial tests performed on the OPC-S material after 28 days curing.

**Figure 4:**
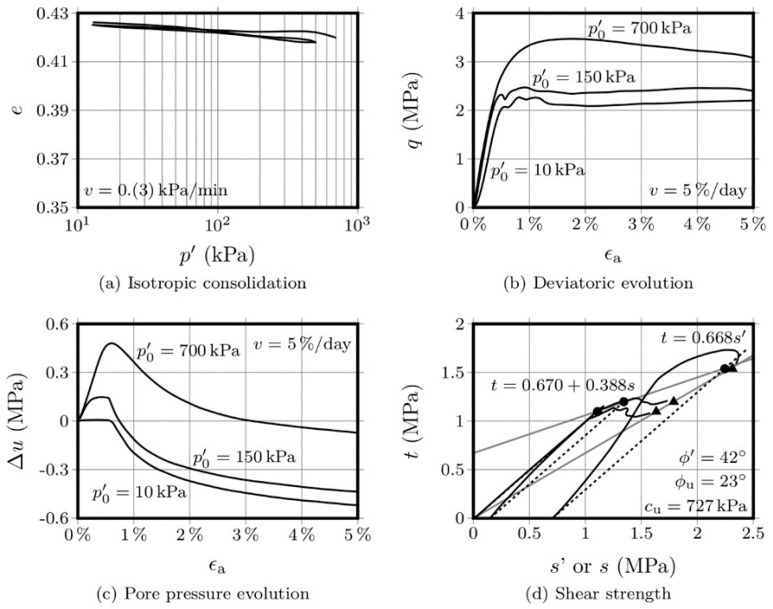
CIU triaxial tests performed on the OPC-S material after 90 days curing.

Figure 3 and Figure 4 show the results obtained with the OPC-S specimens, after 28 and 90 days curing, respectively. Regarding the isotropic compression, it is interesting to observe that the volume change, as measured by the variation of the voids ratio, is significantly lower than the variation presented by the AAC-S specimens. Additionally, the strength increase, after 28 days, was also significantly higher than that obtained with the activated FA materials. This is not surprising, given that the Portland cement has a higher strength increase rate than fly ash-based alkaline cements [1,14]. The influence of the curing period in Portland cement, after 28 days, is negligible, while alkaline cements develop a very significant portion of their final compressive strength after 28 days curing. Therefore, it is not surprising that the variation in voids ratio was very small between the 28 and the 90-day mark, especially when compared with the variation shown by the AAC-S material, for the same curing interval.

A comparison between the stress-strain curves of the 3 materials, after 28 days, shows a higher yield stress in the OPC-S material than that obtained for the AAC-S. However, as mentioned above, the hydration reactions responsible for the Portland cement strength gain mostly develop during the first 28 days, reaching 80 to 90% of its ultimate compressive strength during that period. On the contrary, alkaline cements based on class F fly ash reach only 40% to 60% of its 1-year strength after 90 days [1,14].

The variation of the pore pressure with the axial strain, for both the 28- and 90-day curing periods, showed an increase until the peak deviatoric stress, which is probably a consequence of the residual difference between the peak *q* values obtained for these two curing periods.

The drawing of the failure envelopes allowed to establish an effective friction angle of 43° and an undrained friction angle of 23°, for the 28-day cured specimens. For the 90-day specimens, the effective friction angle was 42° and the undrained friction was 27°. This means that the variation of the frictional component was merely residual, regardless of the drainage conditions. However, the undrained cohesion value increased from 344 kPa to 727 kPa, between the 28 and the 90 days mark, representing 111%. For the 90 days curing period, the OPC-S material showed a higher cohesion than the AAC-S, but the increase between the 28 and 90 days was less pronounced (111% to 310% in the AAC-S material), a clear indication of the slower, yet effective, development of the binding gel in the alkali-activated system.

**Figure 5:**
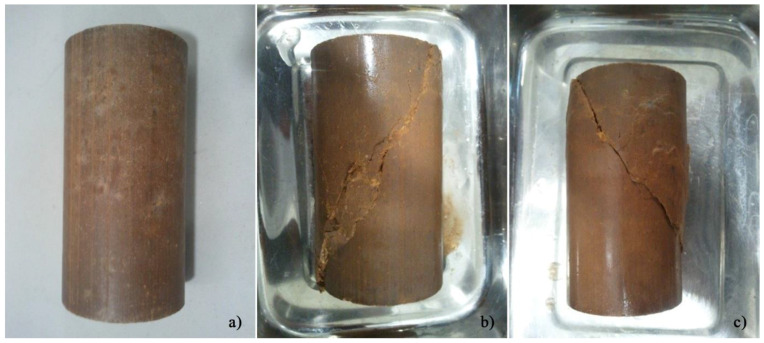
General condition of the OPC-S specimens: (**a**) before testing; (**b**) after testing, with 90 days curing, consolidated to 150 kPa; (**c**) after testing, with 90 days curing, consolidated to 700 kPa.

Figure 5 presents different stages of OPC-S specimens, cured for 90 days. The shear plane is very clear, regardless of the consolidation stress, indicating a similar behaviour already registered for the fly ash-stabilised soil in consolidated undrained testing.

**Figure 6:**
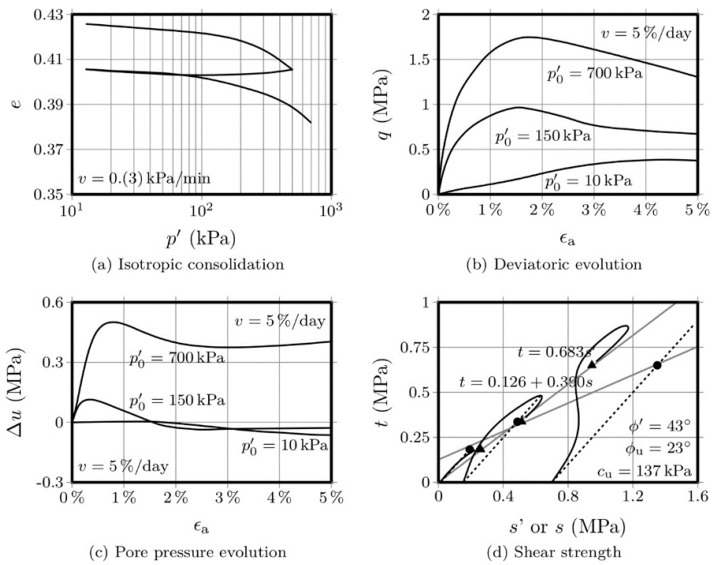
CIU triaxial tests performed on the AAC-S material after 28 days curing.

**Figure 7:**
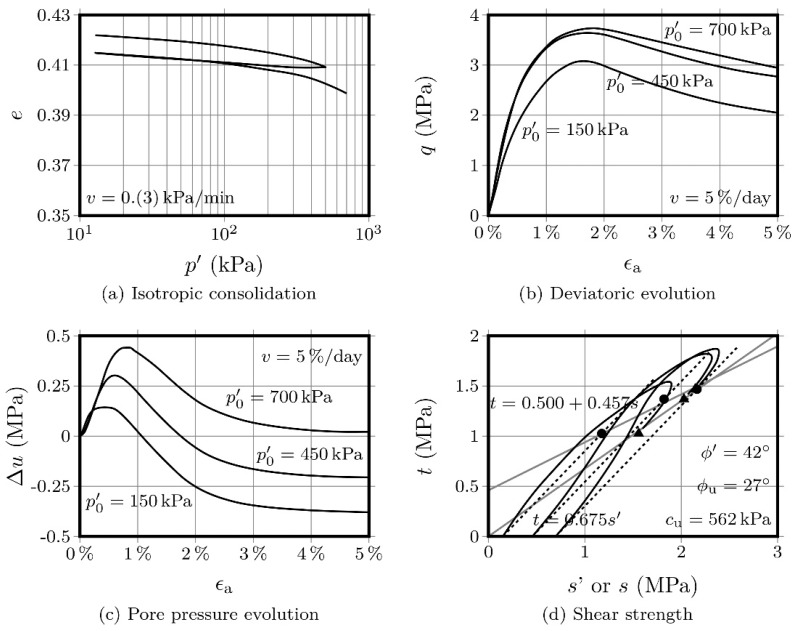
CIU triaxial tests performed on the AAC-S material after 90 days curing.

Triaxial tests’ results for AAC-S are shown in Figure 6 and Figure 7, for specimens with 28- and 90-days curing, respectively. The isotropic compression response shows a clear influence from the curing period, as there is a lower variation of the void ratio for the specimens cured for 90 days, which showed a higher stiffness. Such behaviour is not surprising, as this type of chemical stabilization is strongly influenced by the age of the specimen, since the 28-day interval might not be enough to mature the reaction products that constitute the binding matrix [30]. Therefore, there may be particles not bound by the gel, causing heterogeneity along the soil mass [31].

Regarding the deviatoric stress as a function of the axial strain, there is a clear peak stress, contrary to what was observed for the natural soil, which presented a strain-hardening behaviour. After this peak is reached, there is a decrease, for both curing periods, in deviatoric stress, except for the test specimen with a 10 kPa consolidation stress, after 28 d curing, which was obviously softer at the start of the test. The existence of a peak stress in soil artificially stabilised with activated fly ash is consistent with the trend described by other authors for this type of chemical stabilisation [3,6,16]. The stabilised material showed higher strength and stiffness after the 90-day curing period and, consequently, lower deformation compared with the 28 d curing specimens. Chemical stabilisation tends to show a time dependence, as there is an evolution of the chemical reactions and, consequently, an improvement of the main properties, which sometimes can continue up to 1 year of curing [14].

Similar conclusions can be drawn for the evolution of pore water pressure, with the 90-day curing specimens showing a lower pressure variation, compared with the 28-day specimens, as a result of the development of the binding matrix produced by the chemical reactions. Curiously, pore pressure variation is higher for the AAC-S specimens than for the natural soil, which is a consequence of the lower voids volume in the former, compared with the no-gel, high void volume in the latter.

**Figure 8:**
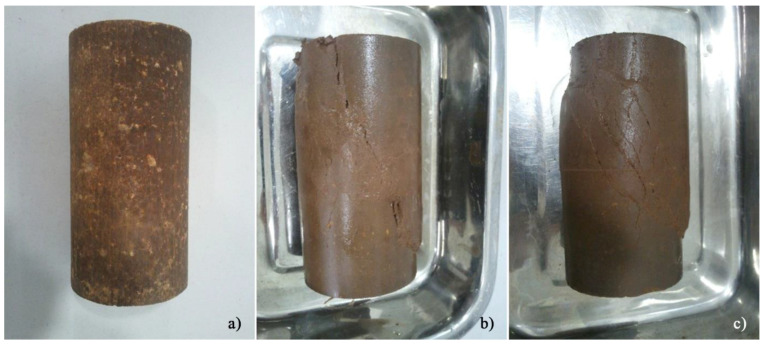
General condition of the AAC-S specimens: (**a**) before testing; (**b**) after testing, with 90 days curing, consolidated to 450 kPa; (**c**) after testing, with 90 days curing, consolidated to 700 kPa.

The failure envelope for both curing periods is also presented, revealing values of *φ*’ = 43°, *φ_u_* = 23° and *c_u_* = 137 kPa, for the 28-day material; and of *φ*’ = 42°, *φ_u_* = 27° and *c_u_* = 562 kPa, for the 90-day material. An increase in the undrained cohesion parameter of 310% clearly indicates a strong influence that curing time can have on the development of the mechanical properties. Finally, Figure 8 compares the overall condition of the specimens before and after being submitted to the respective CIU test. In short, the addition of activated FA produces significant changes in the properties of the natural soil, resulting in an improvement in stiffness and, consequently, in lower deformations.

## 3. Materials and methods

Three types of material were prepared and characterised: natural soil, natural soil stabilised with a conventional binder (Portland cement—OPC), and natural soil stabilised with alkali-activated cements (AAC). The natural soil and the OPC stabilised soil tests were designed to create a solid benchmark to help assess the validity of the AAC-stabilised soil tests, when fly ash was added to the soil particles and subsequently activated with an alkaline solution.

### 3.1. Materials

The soil used in this study was collected near the city of Porto, in the north of Portugal. Several previous studies have focused on the characterisation of this soil [2], and have classified it as a CL-sandy lean clay (based on the Unified Soil Classification System [32], and as belonging to the A-4, silt-clay materials group, according to the AASHTO Soil Classification System [33]. Some geotechnical properties of the soil are presented in Table 1.

The fly ash (FA) was provided by the company PEGOP, responsible for the Portuguese thermo-electric power plant *Central do Pego*. Based on its chemical composition (Table 2), it was classified as a class F fly ash, according to [34]. Portland cement type CEM II/B-L 32.5N was also used as a stabiliser.

All mixtures prepared with AAC or OPC (Table 3) were tested after 28- and 90-days curing. The AAC-soil combination (AAC-S) was prepared with 80 wt.% of soil and 20 wt.% of fly ash, and an activator/FA ratio of 0.96. The activator was a combination of sodium hydroxide (SH) and sodium silicate (SS), with a SS/SH weight ratio of 0.5. The SH was supplied in pellets, with a specific gravity of 2.13 at 20 °C (99 wt.%), which was then dissolved in deionised water to a concentration of 10 molal. The SS presented a unit weight of 1.464 g/cm^3^, a SiO_2_/Na_2_O weight ratio of 2.0 (molar oxide ratio of 2.063), and a Na_2_O concentration in the solution of 13.0%. The OPC-soil combination (OPC-S) was prepared with 95 wt.% of soil and 5 wt.% of OPC.

### 3.2. Methods

The Proctor test on the AAC-S combination was developed with water, instead of activator, to avoid inevitable complications associated with the fast hardening of the soil-stabiliser paste, which could influence the test result. Table 4 shows the compaction properties of the studied materials, namely the dry density and the optimum water content, determined by Proctor tests carried out according to BSi 1377-4 (1990) [35].

Laboratory characterization included the measurement of soil suction and consolidated undrained triaxial tests, either for the natural soil and for both soil-stabiliser combinations, after 28- and 90-days curing in a climatic chamber, at 27 °C and 90% relative humidity.

**Figure 9:**
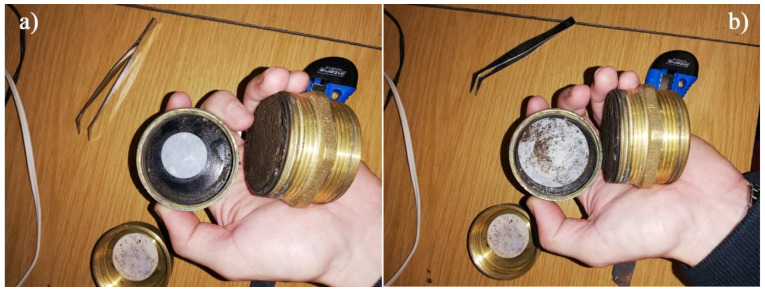
Application of the two concentric paper filters, with diameters of 30 mm (**a**) and 50 mm (**b**).

The variation in the soil matrix suction, which is a function of the degree of saturation, has a direct influence on its shear strength and bearing capacity, as well as on its stiffness [19,20,21]. Soil matrix suction was determined by the filter paper method, following the procedures recommended by ASTM D5298 (2016). Nine cylindrical specimens were prepared for each batch, with 52 mm in diameter and 20 mm in height. The test procedure consisted of measuring the water absorbed by the specimen using an impermeable lateral mould, to prevent any moisture exchange with the atmosphere. After curing, the specimens were oven-dried, at 110 °C, for 24 h, after which two concentric circular filter papers (with 30 mm and 50 mm in diameter) were applied at the base of the specimen (Figure 9), and a 40 mm diameter filter paper was applied at the top.

The filter paper located at the top was then wetted, thus distributing the moisture evenly over the entire top section of the specimen. A different volume of water was added to each of the 9 specimens, to produce 9 different water contents. After wetting, a plastic disc was placed over the 40 mm diameter filter paper to guarantee the contact between the soil and the filter paper. Finally, the specimen was wrapped in plastic tape and stored in a sealed bench container at approximately 22 °C, for 7 days. The wet mass of both the specimen and of each of the three filter papers was then determined and, after drying for 48 h at 110 °C, new mass measurements (now dry) were taken. The results obtained with the 30, 40, and 50 mm papers will be presented in Figure 1. For the AAC-S, two sets of three specimens were fabricated, with one set measured after 28 days and the other after 90 days curing. For the OPC-S, a total of four sets of specimens were fabricated, with three sets measured after 28 days and the remaining set after 90 days curing.

The matric suction values in each specimen were calculated using Equation (1), proposed by ASTM D5298 [23], while Equation (2), proposed by Van Genuchten [36], was adopted to adjust a theoretical curve to the experimental data, of the variation of the volumetric water content (*θ_w_*) as a function of the suction (*ψ*) value. In these two equations, ‘*ω*’ represents the filter paper water content, ‘*θ_s_*’ the saturated volumetric water content, ‘*θ_r_*’ the residual volumetric water content, and *a*, *b* and *c* are fitting parameters. After obtaining these parameters, it is possible to predict the volumetric water content and, thus, the water content, for any suction value, and vice versa [37,38].

(1) logΨ=5.327−ω·7.79

(2) $$\theta_{w}=\theta_{r}+\frac{\theta_{s}-\theta_{r}}{{\left(1+a\cdot \Psi^{b}\right)}^{c}}$$

The consolidated undrained triaxial tests were performed on cylindrical specimens, with 70 mm in diameter and 140 mm high, according to the contents of BSi 1377-8 [39] standard. The 3 materials presented in Table 4 were tested. The test procedure included 3 distinct phases. Initially, a test saturation phase was performed by monotonic increase (over 24 h) of the confining stress and back-pressure, up to 1000 kPa (with a constant 10 kPa difference between them), resulting in values of the Skempton B parameter exceeding 0.97. Subsequently, a consolidation stress was applied. The original soil specimens were consolidated to the confining stresses indicated in Table 5**.** The final shear stage was performed under a constant strain rate of 0.2% per hour.

## 4. Discussion

To the best of our knowledge, this was the first time that soil-water retention curves of a soil stabilised with fly ash-based alkaline cements were drawn. A very interesting advantage of the SWRC of the AAC-S and OPC-S was the insight into the pore structure of these materials, with the wider and ‘softer’ curve presented by the AAC-S indicating a more gradual variation of the internal voids, compared with the OPC-S. Additionally, the changes in the curves between the original and stabilised soil suggest that the binding gel somehow filled (partially) the voids of the original soil, creating a different pore net.

The saturation degree values were not affected by either binder, or by curing periods in excess of 28 days. However, the development of the SWRC is significantly different, especially between the AAC and the OPC binders, but also between the natural and stabilised soil. Such differences, especially between both types of stabilisation, have surely influenced the triaxial tests’ results.

After 28 days curing, the OPC-S material presented higher peak deviatoric stresses than the AAC-S material, and the opposite was observed after 90 days. Such difference is not related to the suction existing in the specimens during the shear stage of the tests, since every specimen went to a previous saturation stage, but probably with the more heterogenous pore volume distribution of the AAC-S, which produced a more compacted and dense structure that could be co-responsible for the strength values shown by this material. This is consistent with the results obtained either after 28 days, when the gel is yet in a early development stage, and should thus present a significantly lower peak stress; or after 90 days, when it presented higher strength levels than the OPC-S.

The other factor contributing to the higher deviatoric stress showed by the AAC-S after 90 days was the quality of its binding gel, which, by then, had further developed and crystalised [14]. The higher deviatoric stress of the OPC-S after 28 days curing was related to the strength gain rate of both binders, which is significantly higher in Portland cement during the first few weeks, stabilising afterwards. On the contrary, fly ash-based alkaline cements take several months to fully develop. The quantification of the relative contribution of these two factors will require further studies.

The similarity of the AAC-S curves after 28- and 90-days curing is also enlightening in terms of the formation of its binding gel. These results indicate that the structure of the gel (i.e., its pore volume distribution) is formed at an early age (not more than 28 days) and, after that, the development of the gel (corroborated by the clear strength increase between 28 and 90 days) is mostly due to its crystallization. The similarity between both retention curves of the OPC-S material also indicates that the gel structure was in-place after 28 days. However, in this case, the gel structure was not only formed after 28 days, but the gel consistency evolution was also complete by then.

## 5. Conclusions

The results obtained throughout the experimental work here presented showed that this type of alternative binder is a valid alternative to OPC-based binders typically used in soil stabilisation. The retention curves of the original and stabilised soil showed consistent differences, enabling a deeper interpretation of the pore volume structure of each solution:

The initial air-entry values are very different for the soil and respective stabilisation solutions, despite the similar saturation water contents. The natural soil’s high air-entry value indicates that it remains saturated until suction values much higher than those observed for the OPC-S and AAC-S.

The soil-gel structure is already formed after 28 days, resulting in similar curves for both curing periods. This is particularly surprising in the case of the alkaline cement gel, known to have a development period longer than 28 days.

The higher deviatoric stresses shown by the AAC-S after 90 days, compared with the OPC-S values for the same period, are not related to the evolution of the pore volume structure but, instead, to the development and crystallization of the gel.

The matrix suction is clearly influenced by the type of chemical stabilisation, regarding the shape of the curves and respective air-entry values, suggesting that the results obtained open the possibility of using the retention curve analysis to assess the effectiveness of the stabilisation process, when based on alkaline cements.

## Figures and Tables

**Table 1:** Geotechnical properties of the soil [2].

Property	Value
Sand (%)	39.3
Fines (%)	60.7
Liquid limit (%)	28
Plasticity limit (%)	19
Specific gravity	2.54

**Table 2:** Chemical composition of the fly ash.

Element	wt.%
Al_2_O_3_	20.82
CaO	1.84
Cr_2_O_3_	0.02
Fe_2_O_3_	8.62
K_2_O	2.75
MgO	1.94
MnO	0.06
Na_2_O	1.08
SiO_2_	60.00
SO_3_	0.88
Outros	1.64
L.O.I.	0.34

**Table 3:** Composition of the materials tested.

Material	Dry Soil (wt.%)	Dry FA (wt.%)	Dry OPC (wt.%)	10 m Activator Content (wt.%)	Water Content (wt.%)
Original soil	100	-	-	-	14.4 ^(^*^)^
OPC-S	95	-	5	-	14.5 ^(^*^)^
AAC-S	80	20	-	19.2 ^(**)^	-

^(^*^)^ Based on the results of the Proctor tests (see Table 4); ^(^**^)^ Higher than the value obtained with the Proctor tests (13.0%, see Table 4), to compensate the Na_2_O molecules that weren’t included in the liquid phase (water) of the Proctor test.

**Table 4:** Compaction parameters of the chemically stabilised materials, as determined by Proctor tests.

Material	Maximum Dry Unit Weight (g/cm^3^)	Optimum Water Content (%)
Original soil	1.81	14.4
OPC-S	1.82	14.5
AAC-S	1.80	13.0

**Table 5:** Consolidation stresses for the triaxial tests.

Material	Consolidation Stress (kPa)		
	Test 1	Test 2	Test 3
Original soil	50	100	200
OPC-S 28 days	10	450	700
OPC-S 90 days	10	150	700
AAC-S 28 days	10	150	700
AAC-S 90 days	150	450	700
